# 1,25-dihydroxyvitamin D_3_ suppresses lipopolysaccharide-induced interleukin-6 production through aryl hydrocarbon receptor/nuclear factor-κB signaling in oral epithelial cells

**DOI:** 10.1186/s12903-019-0935-x

**Published:** 2019-11-04

**Authors:** Hao Li, Wei Li, Qi Wang

**Affiliations:** 1 Department of Prosthodontics, the Affiliated Hospital of Stomatology, Guangxi Medical University, 10 Shuangyong Road, Nanning, 530021 People’s Republic of China; 2 State Key Laboratory of Oral Diseases, West China Hospital of Stomatology, Sichuan University, 14 3rd Section S Renmin Road, Chengdu, 610041 People’s Republic of China; 3 Loma Linda University School of Dentistry, 24876 Taylor Street, Loma Linda, CA 92354 USA

**Keywords:** Oral epithelial cells, 1,25-dihydroxyvitamin D_3_, Aryl hydrocarbon receptor, Nuclear factor-κB, Interleukin-6

## Abstract

**Background:**

Antiinflammatory effect of 1,25-dihydroxyvitamin D_3_ (1,25D3) has been reported in periodontitis, but the exact mechanisms remain unclear. Oral epithelial cells are recently highlighted as an important regulator of inflammation in this disease. This in vitro study was established to investigate the effect of 1,25D3 on key proinflammatory cytokine IL-6 production and aryl hydrocarbon receptor (AhR)/nuclear factor-κB (NF-κB) signaling in oral epithelial cells upon the stimulation of lipopolysaccharide (LPS) from periodontal pathogens.

**Methods:**

OKF6/TERT-2 oral keratinocytes were incubated with LPS and different concentrations of 1,25D3, and levels of IL-6 production were determined using enzyme-linked immunosorbent assay (ELISA). Expression of vitamin D receptor (VDR), and activation of AhR was examined using western blot analysis, and phosphorylation of NF-κB was detected using cell-based protein phosphorylation ELISA.

**Results:**

1,25D3 inhibited LPS-induced IL-6 overexpression in OKF6/TERT-2 cells. Additionally, 1,25D3 increased VDR expression and AhR activation, and repressed NF-κB phosphorylation. Furthermore, 1,25D3 suppressed IL-6 expression and enhanced VDR expression and regulated AhR/NF-κB signaling activation in a dose-dependent manner after 48 h treatment.

**Conclusions:**

These results suggest that 1,25D3 may inhibit LPS-induced IL-6 overexpression in human oral epithelial cells through AhR/NF-κB signaling. Our findings may provide an explanation for the antiinflammatory effect and therapeutic benefit of 1,25D3 in periodontitis.

## Background

Periodontitis is characterized by the loss of periodontal attachment, including periodontal ligament and alveolar bone, caused by the host immune response to bacterial insult [1]. Lipopolysaccharide (LPS) is a key pathogenic component of periodontal pathogens [2]. It has been broadly reported to induce host cells like oral epithelial cells to produce a wide range of proinflammatory cytokines, including tumor necrosis factor-α (TNF-α), interleukin-6 (IL-6) and interleukin-8 (IL-8), promoting periodontitis progression [3, 4]. As a crucial stimulator of alveolar bone resorption, IL-6 is overexpressed in the periodontium in periodontitis and greatly responsible for periodontal destruction [5].

Oral epithelial cells are recently highlighted as a critical regulator of inflammation in periodontitis [6, 7]. In response to LPS, they can produce a range of inflammation-related proteins, leading to potent inflammatory responses in periodontal diseases. Upon LPS stimulation, nuclear factor-κB (NF-κB), a key regulator of inflammation-related gene transcription, can be activated in oral epithelial cells, resulting in the production of proinflammatory cytokines, such as IL-6 [8, 9]. Regulating the inflammatory response in oral epithelial cells is considered to be a potential strategy for periodontitis treatment.

An increasing amount of literature demonstrates the antiinflammatory effect of steroid hormone 1,25-dihydroxyvitamin D_3_ (1,25D3) in different inflammatory diseases, including Crohn’s disease and diabetes [10, 11]. It is the active form of vitamin D_3_, and possesses little adverse effects in clinical application [12]. It acts on vitamin D receptor (VDR), and subsequently exerts immunomodulatory functions in many epithelial cells, including oral epithelial cells [13, 14]. Current research has shown that supplementation with different forms of 1,25D3 regulates the expression of inflammatory cytokines, such as TNF-α and IL-8, in oral epithelial cells, and reduces alveolar bone loss in periodontitis [15, 16]. These findings indicate the therapeutic effect of 1,25D3 in periodontitis and the regulation of inflammatory responses by 1,25D3 in oral epithelial cells. However, the exact mechanisms remain unclear.

Aryl hydrocarbon receptor (AhR), a nuclear transcription factor, has been reported to play an important role in inflammatory modulation during recent years [17]. Upon binding to its ligand, AhR translocates from the cytoplasm into the nucleus, and then activates the transcription of target genes including cytochrome P450 1A1 (CYP1A1) and downstream inflammatory cytokines. AhR activation can improve immune homeostasis in epithelial cells, such as intestinal epithelial cells [18], and its activation can be observed in oral epithelial cells upon stimulation with oral commensal bacterium *Streptococcus mitis* [19]. Current research has shown the crosstalk between AhR and NF-κB signaling in chronic inflammatory response of bronchial epithelial cells [20]. Additionally, activation of AhR signaling can be enhanced by 1,25D3 in different immune cells like monocytic cells and kidney epithelium-derived cells [21]. These findings suggest that 1,25D3 might modulate inflammatory response in periodontitis through regulating AhR/NF-κB signaling. In this report, we cultivated OKF6/TERT-2 oral keratinocytes with LPS and different concentrations of 1,25D3, and examined the changes of IL-6 expression and AhR/NF-κB signaling activation.

## Methods

### Cell culture

Human oral keratinocytes (OKF6/TERT-2), kindly provided by Dr. J. Rheinwald (Harvard University, Boston, MA), were cultured in accordance with the protocols as described previously [22]. The cells were plated at 1 × 10^5^/well in 96-well plates in keratinocyte serum-free medium containing *Porphyromonas gingivalis* (*P. gingivalis*) LPS (Sigma-Aldrich, St. Louis, MO) (1 μg/ml) and 1,25D3 (Sigma-Aldrich, St. Louis, MO) (0 nM, 10 nM, 20 nM, or 30 nM), and incubated for 6 h, 12 h, 24 h or 48 h [23]. 1,25D3 was dissolved in ethanol, and ethanol was chosen as vehicle (ethanol volume was 0.1% of culture medium solution).

### Enzyme-linked immunosorbent assay (ELISA) for IL-6

Culture supernatants were collected at 6 h, 12 h, 24 h and 48 h after LPS and 1,25D3 incubation, and IL-6 concentrations were detected using a commercially available ELISA kit (R&D Systems, Minneapolis, MN) according to the manufacturer’s instructions [22]. The solution absorbance was measured at 450 nm using a microplate reader (Thermo Fisher Scientific, Waltham, MA) for quantity calculation.

### Western blot analysis

At every predetermined time point, cultured cells in each group were harvested. Total protein extracts from cultured cell lysates were prepared in RIPA buffer with 1% proteinase and 1% phosphate inhibitors, and the expression of VDR, AhR and CYP1A1 was examined using western blot analysis. The protocol of western blot analysis was performed as previously reported [24]. Protein samples were subjected to electrophoresis on 8% sodium dodecyl sulfate-polyacrylamide gel, and transferred to polyvinylidene difluoride membranes. Afterwards, the membranes were probed with primary monoclonal antibodies against VDR (1:200), AhR (1:500), CYP1A1 (1:300) and glyceraldehyde 3-phosphate dehydrogenase (GAPDH) (1:500), and then incubated with horseradish peroxidase (HRP)-conjugated secondary antibodies (1:2000). Antibody complexes were detected using the SuperSignal West Pico Chemiluminescent Substrate System. All of the antibodies were from Santa Cruz Biotechnology (Santa Cruz, CA).

### Cell-based enzyme-linked immunosorbent assay for NF-κB

Percentage of NF-κB p65 phosphorylation was measured using commercially available cell-based protein phosphorylation ELISA kits (R&D Systems, Minneapolis, MN) as previously reported [22]. At 6 h, 12 h, 24 h and 48 h after LPS and 1,25D3 incubation, OKF6/TERT-2 cells in 96-well plates were fixed by addition of 4% formaldehyde, and incubated with monoclonal anti-total NF-κB p65 or anti-phosphorylated NF-κB p65. Then, the cells were incubated with HRP-conjugated secondary antibody at a dilution of 1:1000 [22]. The absorbance of each well was detected at a wavelength of 570 nm using a multifunctional microplate reader (Thermo Fisher Scientific, Waltham, MA), and afterward the percentage of NF-κB p65 phosphorylation was calculated.

### Statistical methods

All experiments were performed in triplicate with independent samples. The results were expressed as the mean ± SD, and statistical significance was analyzed by one-way ANOVA followed by SNK-*q* multiple comparisons. Pearson’s correlation coefficient was used to detect the correlation between IL-6, VDR, AhR or CYP1A1 levels and 1,25D3 concentrations, and between phosphorylation of NF-κB p65 and 1,25D3 concentrations, when cells were treated with LPS and 1,25D3 for 48 h. SPSS 20.0 software (SPSS Inc., Chicago, IL) was used for statistical analysis. A *p*-value < 0.05 in two tailed analysis was considered to be statistically significant.

## Results

### Effects of 1,25D3 on LPS-induced IL-6 production

**Fig. 1:**
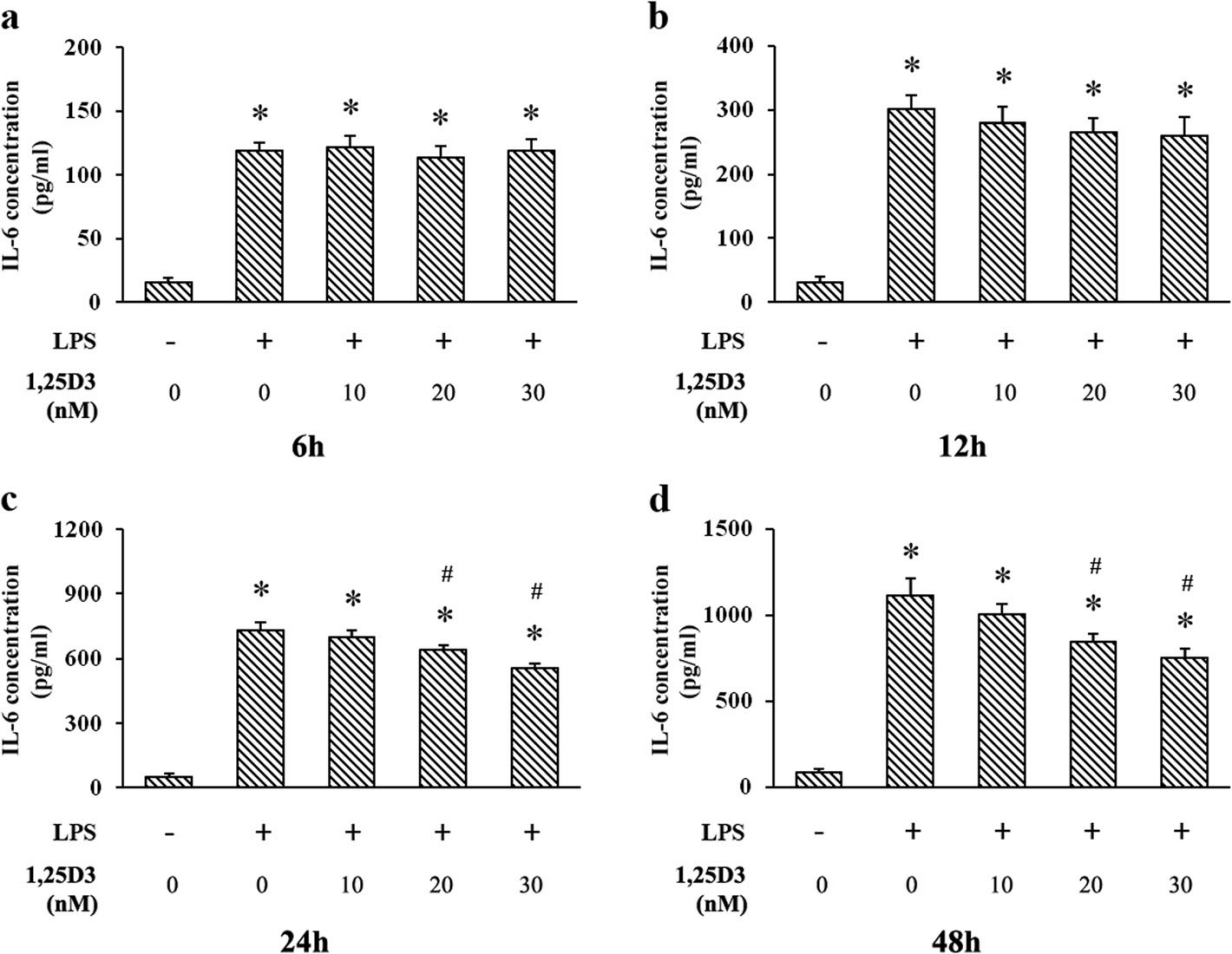
IL-6 production in LPS-treated oral epithelial cells was detected using ELISA. OKF6/TERT-2 cells were cultured for 6 h (**a**), 12 h (**b**), 24 h (**c**) or 48 h (**d**), in the presence of LPS (1 μg/ml) and 1,25D3 (0, 10, 20, or 30 nM). 1,25D3 at 20 nM and 30 nM significantly inhibited LPS-induced IL-6 production after 24 h and 48 h treatment. * *p* < 0.05, significantly different vs. control group. ^#^ *p* < 0.05, vs. LPS control

Culture supernatants of OKF6/TERT-2 cells were detected using ELISA at every predetermined time point. We found that LPS markedly upregulated IL-6 production at each time point (Fig. 1). 1,25D3 treatment was not effective on LPS-induced IL-6 expression after 6 h or 12 h, but 1,25D3 at 20 nM and 30 nM effectively decreased the IL-6 production after 24 h and 48 h (Fig. 1). The correlation between IL-6 levels and 1,25D3 concentrations after 48 h treatment was statistically significant (r = 0.985; *p* < 0.05).

### Effects of 1,25D3 on VDR expression

**Fig. 2:**
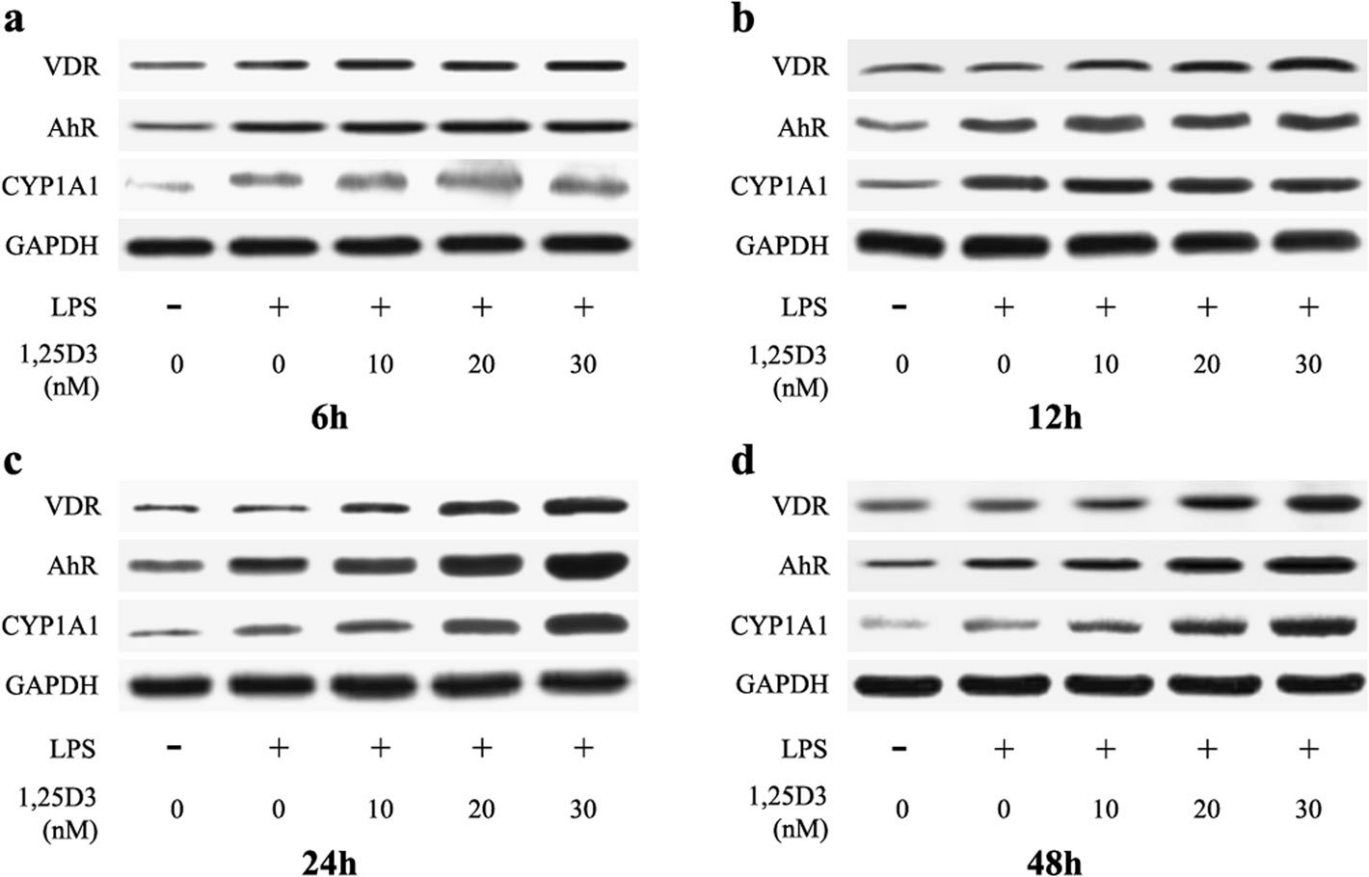
VDR, AhR and CYP1A1 expressions in LPS-treated oral epithelial cells were examined using western blot analysis. OKF6/TERT-2 cells were cultured for 6 h (**a**), 12 h (**b**), 24 h (**c**) or 48 h (**d**), in the presence of LPS (1 μg/ml) and 1,25D3 (0, 10, 20, or 30 nM)

**Fig. 3:**
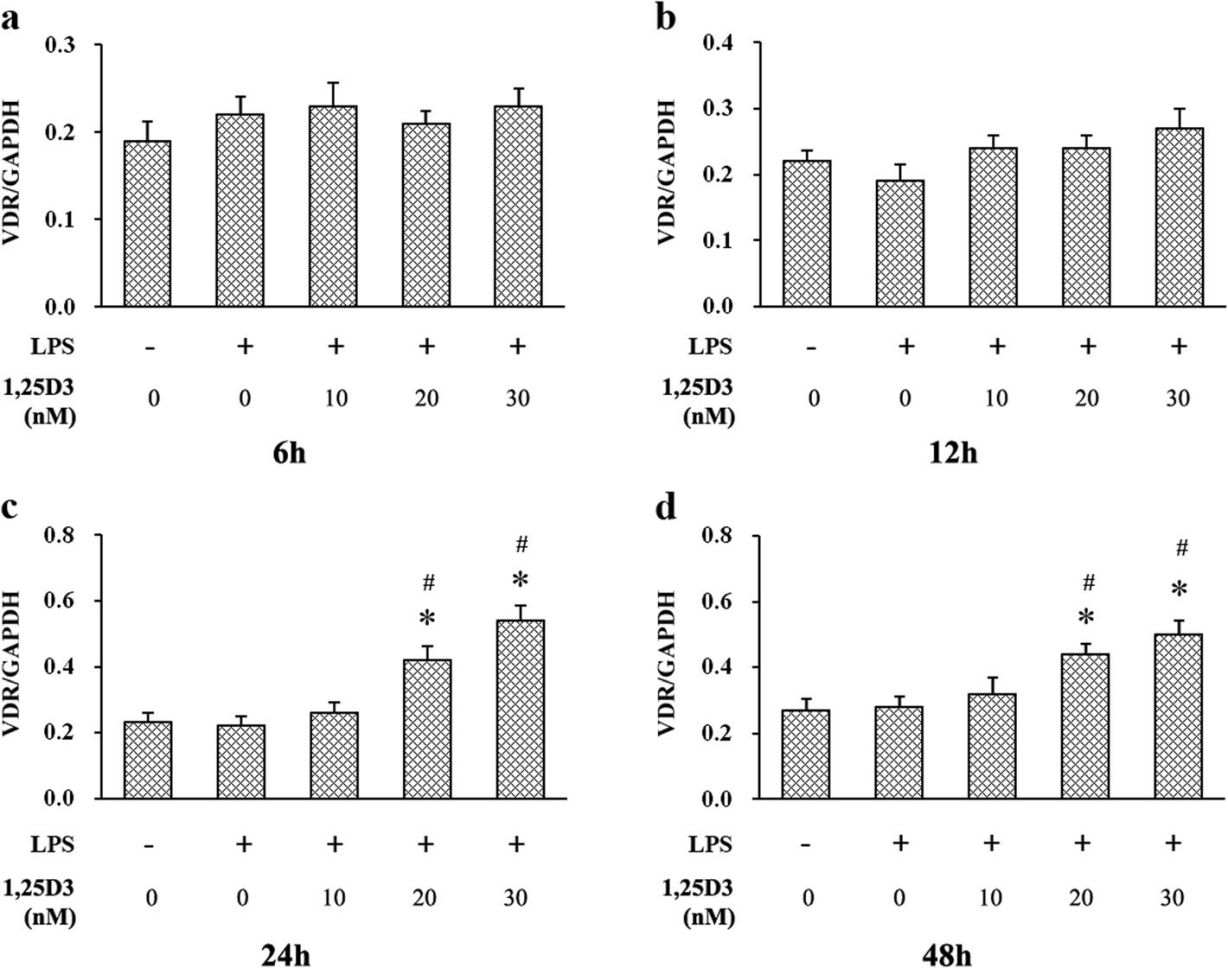
Relative expression of VDR in LPS-treated oral epithelial cells was calculated after western blot analysis. OKF6/TERT-2 cells were cultured for 6 h (**a**), 12 h (**b**), 24 h (**c**) or 48 h (**d**), in the presence of LPS (1 μg/ml) and 1,25D3 (0, 10, 20, or 30 nM). 1,25D3 at 20 nM and 30 nM significantly increased VDR expression after 24 h and 48 h treatment. * *p* < 0.05, significantly different vs. control group. ^#^ *p* < 0.05, vs. LPS control

It is well established that 1,25D3 regulates biological events by binding to VDR [25], and increased VDR expression has usually been observed in 1,25D3 activation [26–28]. We examined VDR expression in OKF6/TERT-2 cells using western blot analysis. As shown in Figs. 2 and 3, no significant difference of VDR expression was observed among all groups after 6 h or 12 h incubation. However, cells cultured in the medium containing 20 nM and 30 nM 1,25D3 showed significantly enhanced VDR expression after 24 h and 48 h, compared with those in the medium with 0 nM and 10 nM 1,25D3. The correlation between VDR levels and 1,25D3 concentrations after 48 h treatment was statistically significant (r = 0.949, *p* < 0.05).

### Effects of 1,25D3 on LPS-induced AhR and CYP1A1 expression

**Fig. 4:**
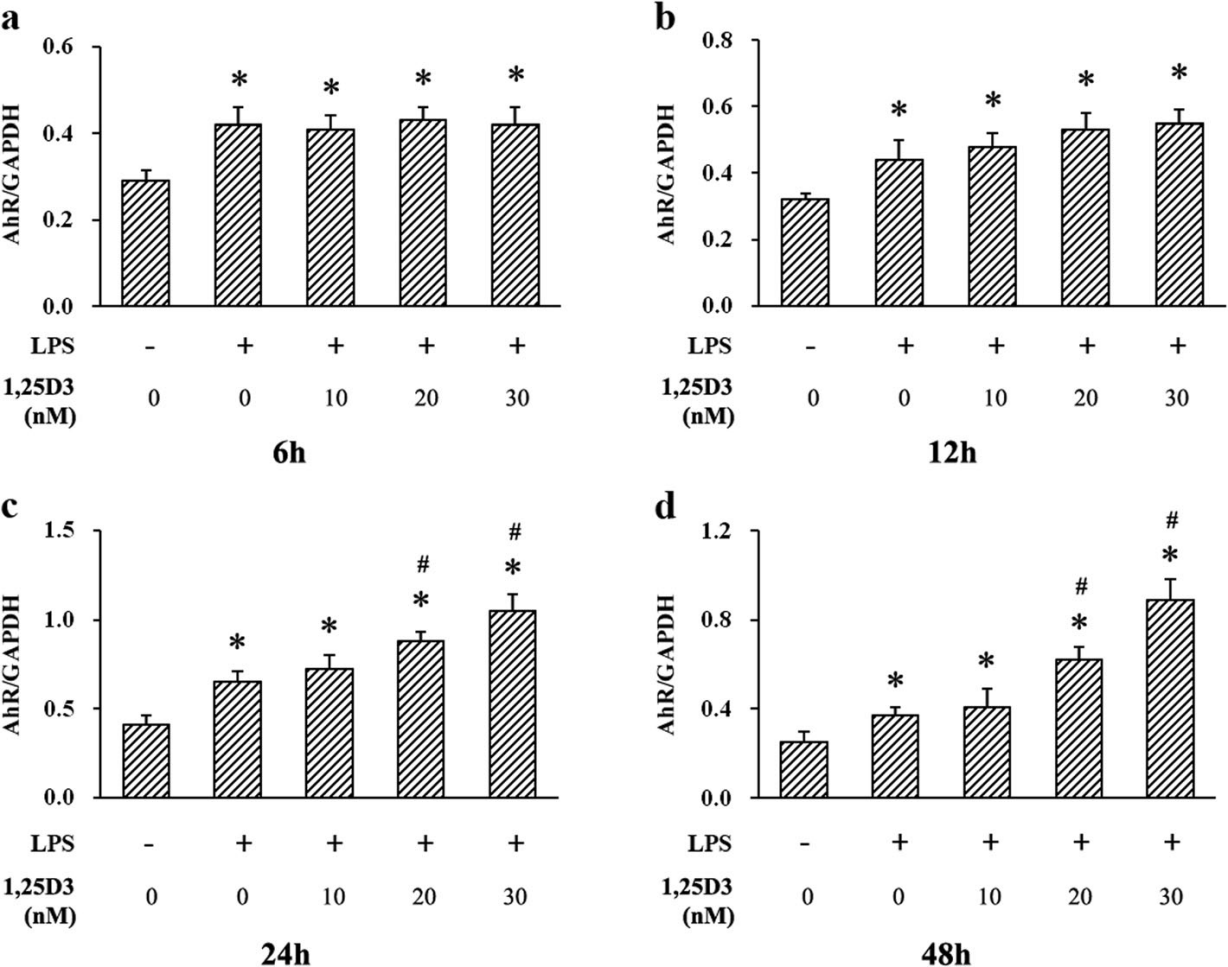
Relative expression of AhR in LPS-treated oral epithelial cells was calculated after western blot analysis. OKF6/TERT-2 cells were cultured for 6 h (**a**), 12 h (**b**), 24 h (**c**) or 48 h (**d**), in the presence of LPS (1 μg/ml) and 1,25D3 (0, 10, 20, or 30 nM). 1,25D3 at 20 nM and 30 nM significantly enhanced LPS-induced AhR expression after 24 h and 48 h treatment. * *p* < 0.05, significantly different vs. control group. ^#^ *p* < 0.05, vs. LPS control

To further investigate the potential mechanism of the suppression of LPS-induced IL-6 upregulation by 1,25D3, we examined the expression of AhR and CYP1A1 in OKF6/TERT-2 cells using western blot analysis. As shown in Figs. 2 and 4, cultivation of OKF6/TERT-2 cells with LPS greatly enhanced AhR expression after 6 h, 12 h, 24 h and 48 h. No significant difference of AhR expression was observed among all LPS-treated cells for 6 h and 12 h. However, cultivation with 20 nM and 30 nM 1,25D3 for 24 h and 48 h obviously increased LPS-induced AhR expression. The correlation between AhR levels and 1,25D3 concentrations after 48 h treatment was statistically significant (r = 0.872; *p* < 0.05).

**Fig. 5:**
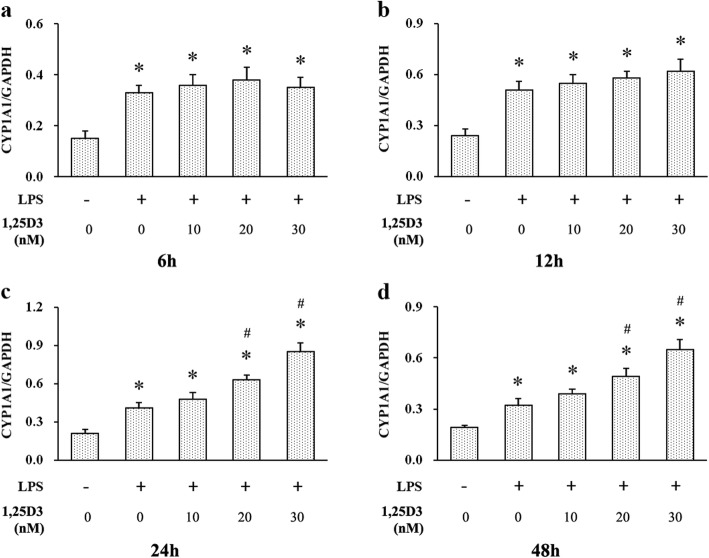
Relative expression of CYP1A1 in LPS-treated oral epithelial cells was calculated after western blot analysis. OKF6/TERT-2 cells were cultured for 6 h (**a**), 12 h (**b**), 24 h (**c**) or 48 h (**d**), in the presence of LPS (1 μg/ml) and 1,25D3 (0, 10, 20, or 30 nM). 1,25D3 at 20 nM and 30 nM significantly increased LPS-induced CYP1A1 expression after 24 h and 48 h treatment. * *p* < 0.05, significantly different vs. control group. ^#^ *p* < 0.05, vs. LPS control

At every indicated time point, LPS enhanced CYP1A1 expression in OKF6/TERT-2 cells (Figs. 2 and 5). 1,25D3 treatment for 6 h and 12 h was not effective on LPS-induced CYP1A1 expression in cells, whereas cells with 20 nM and 30 nM 1,25D3 incubation for 24 h and 48 h showed increased CYP1A1 expression, compared with those with 0 nM and 10 nM 1,25D3 (Figs. 2 and 5). After 48 h, the correlation between CYP1A1 levels and 1,25D3 concentrations was statistically significant (r = 0.923; *p* < 0.05).

### Effects of 1,25D3 on LPS-upregulated NF-κB phosphorylation

**Fig. 6:**
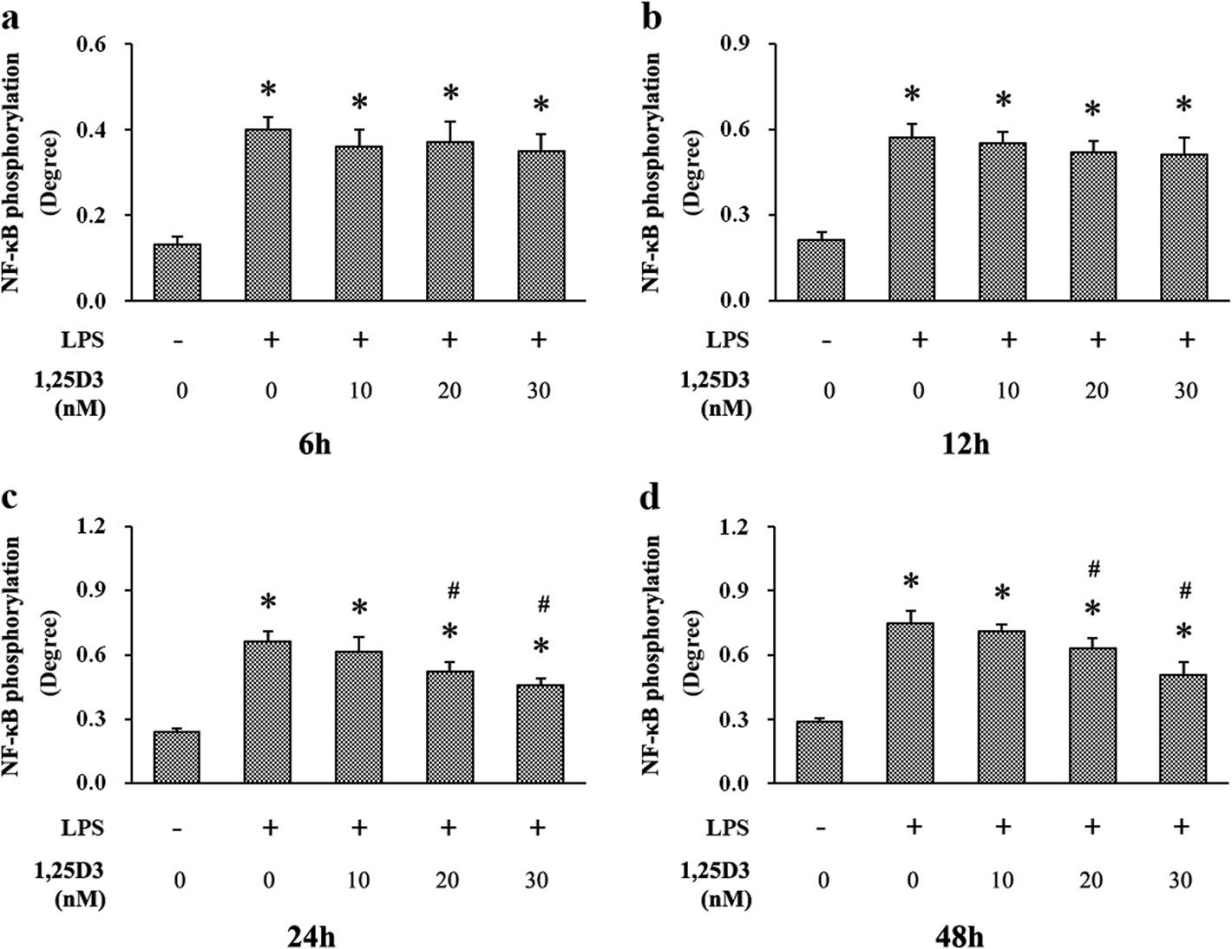
Phosphorylation of NF-κB in LPS-treated oral epithelial cells was examined using cell-based protein phosphorylation ELISA. OKF6/TERT-2 cells were cultured for 6 h (**a**), 12 h (**b**), 24 h (**c**) or 48 h (**d**), in the presence of LPS (1 μg/ml) and 1,25D3 (0, 10, 20, or 30 nM). 1,25D3 at 20 nM and 30 nM significantly suppressed LPS-induced NF-κB phosphorylation after 24 h and 48 h treatment. * *p* < 0.05, significantly different vs. control group. ^#^ *p* < 0.05, vs. LPS control

We measured NF-κB p65 activation using cell-based protein phosphorylation ELISA kits. All cells stimulated with LPS exhibited enhanced phosphorylation of NF-κB p65 at every predetermined time point, compared with those unstimulated cells (Fig. 6). Different concentrations of 1,25D3 treatment for 6 h and 12 h did not significantly decrease NF-κB p65 phosphorylation under LPS stimulation. However, after 24 h and 48 h incubation, 20 nM and 30 nM 1,25D3 showed obvious suppressive effect on LPS-induced NF-κB p65 phosphorylation in cells (Fig. 6). The correlation between NF-κB p65 phosphorylation levels and 1,25D3 concentrations after 48 h treatment was statistically significant (r = 0.861; *p* < 0.05).

## Discussion

To date, accumulating evidence has shown the protective function of 1,25D3 in inflammatory diseases [10, 11]. However, in clinical studies, the effect of vitamin D on periodontal diseases still remains controversial. Some human research showed that lower serum levels of 25-hydroxyvitamin D_3_, the stable form of 1,25D3 in the body, were significantly related to periodontitis [29, 30]. However, several investigators demonstrated that serum vitamin D levels or vitamin D supplementation did not seem to be associated with periodontal status [16, 31]. These different observations may partly be attributed to wrong study designs, poorly paired case-controls, and short follow-up duration, etc. Compared with human clinical studies, research on in vitro models has less uncontrollable variables, so different periodontal cells, including oral epithelial cells, cocultured with the virulence factors from periodontal pathogens, have been used to establish in vitro models for investigating periodontal diseases.

LPS from periodontal pathogens, including *P. gingivalis*, is one of the major virulence factors in periodontitis. It can induce the overexpression of different proinflammatory cytokines detrimental to periodontal tissues, such as IL-6 [4]. Former studies have shown that IL-6 is a potent stimulator of periodontal bone resorption [6]. Downregulated production of IL-6 is correlated with attenuation of inflammatory response in periodontitis [32]. In this study, LPS markedly increased IL-6 expression levels in OKF6/TERT-2 cells at each time point, indicating the proinflammatory effect of LPS on oral epithelial cells. Additionally, 1,25D3 treatment effectively decreased the IL-6 production after 24 h and 48 h, though it was not effective after 6 h or 12 h, suggesting the inhibitory effect of 1,25D3 on inflammatory response in oral epithelial cells. 1,25D3 is a recently discovered determinant of immune response in inflammatory diseases, such as Crohn’s disease and diabetes [10, 11]. It has been reported that elevated levels of the stable form of 1,25D3 in serum exhibit positive effects on the regulation of excessive inflammatory states in periodontitis [33]. 1,25D3 application also suppresses the production of IL-6 by monocytes after LPS stimulation [33].

1,25D3 regulates multiple physiological processes, including immunity, through binding to VDR, a key protein in 1,25D3-induced signaling [23]. After binding to 1,25D3, VDR interacts with the heterodimer partner retinoid X receptor, and modulates downstream inflammation-related signaling [13]. 1,25D3 treatment has been found to enhance VDR expression in different epithelial cells, such as colonic epithelial cells and pulmonary artery epithelial cells, ameliorating excessive inflammatory response [14, 34]. Here, we observed that VDR protein expression was increased in cells after 24 h and 48 h 1,25D3 incubation, indicating that 1,25D3 could significantly exert its biological function at these time points. As VDR expression was enhanced, IL-6 production was decreased in cells with LPS stimulation, suggesting the inhibitory effect of 1,25D3 on LPS-induced IL-6 production in oral epithelial cells.

To investigate the underlying mechanism of the effect of 1,25D3 on IL-6 production in oral epithelial cells, we detected AhR and its downstream protein CYP1A1 in OKF6/TERT-2 cells using western blot analysis. We found that exposure of OKF6/TERT-2 cells to LPS increased AhR expression at every predetermined time point. In addition, 1,25D3 treatment for 24 h and 48 h enhanced LPS-induced AhR expression and decreased IL-6 production. These observations suggest that 1,25D3 may inhibit LPS-induced IL-6 production through activating AhR signaling. Consistent with our study, LPS exposure can increase AhR expression in other immune cells, such as dendritic cells [35]. Production of proinflammatory cytokines in intestinal epithelial cells can be attenuated by upregulation of AhR activity [36]; and enhanced AhR activation alleviates inflammatory diseases, including colitis, in experimental animals [36, 37].

CYP1A1 activity is often used to assess the level of AhR activation [38]. After binding to the AhR nuclear translocator, AhR translocates from the cytoplasm to the nucleus to modulate the expression of CYP1A1 [38]. We found an increase in CYP1A1 expression in cells treated with LPS, compared with vehicle controls, suggesting the activation of AhR signaling by LPS. Previous research has shown a decline in the efficacy of immune system and excessive inflammatory response in AhR knockout mice [36]. Activation of AhR signaling is essential to maintain immune homeostasis in colon epithelial cells under the condition of LPS stimulation [36]. After 1,25D3 incubation for 24 h and 48 h, LPS-induced CYP1A1 production in cells was enhanced and IL-6 level was decreased. These results indicate that downregulation of IL-6 production by 1,25D3 may be attributed to the activation of AhR signaling. In monocytic cells, 1,25D3 augments the activation of AhR signaling and induction of CYP1A1 by AhR agonist [21]. VDR can interact with AhR signaling, and regulates CYP1A1 transcription [21].

NF-κB signaling is central in regulation of inflammatory responses in many diseases through binding to κB-sites in the promoter region of a variety of proinflammatory genes, including several cytokines [39]. In periodontitis, NF-κB p65 can be significantly activated by *P. gingivalis* LPS, and its phosphorylation is closely associated with IL-6 production and periodontal damage [22, 40]. Different reports have shown the inhibition of NF-κB p65 activation can reduce inflammatory process and attenuate tissue destruction in the periodontium [41, 42]. Here, we examined NF-κB p65 activation using cell-based protein phosphorylation ELISA. We also observed that NF-κB p65 phosphorylation and IL-6 production were enhanced in cells stimulated with LPS at each time point, compared with unstimulated cells. Moreover, NF-κB p65 phosphorylation and IL-6 production were decreased after 24 h and 48 h 1,25D3 treatment, suggesting the suppression of LPS-induced IL-6 expression by 1,25D3 through NF-κB p65. Furthermore, the inhibitory effect of 1,25D3 accompanied with enhanced AhR activation was found in cells, suggesting that the effect of 1,25D3 on IL-6 production may be regulated through AhR/NF-κB signaling. Previous studies have shown that AhR signaling can inhibit NF-κB activity and IL-6 induction to attenuate inflammatory response in bone marrow stromal cells, which are also important cells in periodontal tissues [43]. In different cells, such as bronchial epithelial cells, AhR signaling not only represses NF-κB activation by strong NF-κB activator LPS, but also reduces the binding of NF-κB to its cognate enhancer sequence, leading to amelioration of inflammatory responses [20, 44].

A variety of signaling pathways are implicated in inflammatory modulation by 1,25D3. Previous research has shown that 1,25D3 negatively regulates the expression of Toll-like receptor (TLR) 2 and 4, the specific receptors for *P. gingivalis* LPS, in human monocytes stimulated by LPS [45]. As the upstream proteins of NF-κB signaling, TLR 2 and 4 can interact with adaptor molecule myeloid differentiation primary response gene 88 upon LPS stimulation, and subsequently activates NF-κB pathway, leading to the production of inflammatory cytokine, such as IL-6 [46, 47]. A report on dendritic cells has also shown the regulation of AhR on TLR signaling through TNF receptor-associated factor 6 after LPS conditioning [48]. These studies suggest that the inhibitory effect of 1,25D3 on NF-κB activation and inflammatory cytokine expression in oral epithelial cells treated with LPS may also be associated with the crosstalk between AhR and TLR signalings. However, further experiments are required, such as detection of TLR and NF-κB signalings in AhR or CYP1A1 knockdown periodontal cells in the periodontitis environment after 1,25D3 treatment, to fully address the interaction between different pathways and the precise mechanisms of 1,25D3 in periodontitis attenuation.

## Conclusions

In conclusion, we observed that 1,25D3 inhibited LPS-upregulated IL-6 production in OKF6/TERT-2 cells. Additionally, 1,25D3 increased AhR and CYP1A1 expression, and suppressed the activation of NF-κB. These effects of 1,25D3 could be found in a dose-dependent manner after 48 h treatment. The results suggest that 1,25D3 may suppress LPS-induced IL-6 overexpression in oral epithelial cells through AhR/NF-κB signaling. The present study extends the previous findings on the anti-inflammatory functions of 1,25D3 in periodontitis.

## Data Availability

The datasets used and/or analyzed during the current study are available from the corresponding author on reasonable request.

## Abbreviations

1,25D3: 1,25-dihydroxyvitamin D_3_
AhR: aryl hydrocarbon receptor
CYP1A1: cytochrome P450 1A1
ELISA: enzyme-linked immunosorbent assay
GAPDH: glyceraldehyde 3-phosphate dehydrogenase
IL-6: interleukin-6
LPS: lipopolysaccharide
NF-κB: nuclear factor-κB
TLR: Toll-like receptor
VDR: vitamin D receptor

## References

[1] Herath T, Wang Y, Seneviratne C, Lu Q, Darveau R, Wang C, Jin L (2011). Porphyromonas gingivalis lipopolysaccharide lipid a heterogeneity differentially modulates the expression of IL-6 and IL-8 in human gingival fibroblasts. J Clin Periodontol.

[2] Jain S, Darveau R. Contribution of Porphyromonas gingivalis lipopolysaccharide to periodontitis. Periodontol 2000. 2010; 54:53–70. 10.1111/j.1600-0757.2009.00333.x PMC2943730 20712633

[3] Imai H, Fujita T, Kajiya M, Ouhara K, Miyagawa T, Matsuda S, Shiba H, Kurihara H (2014). Amphotericin B down-regulates Aggregatibacter actinomycetemcomitans-induced production of IL-8 and IL-6 in human gingival epithelial cells. Cell Immunol.

[4] Ohno T, Yamamoto G, Hayashi J, Nishida E, Goto H, Sasaki Y, Kikuchi T, Fukuda M, Hasegawa Y, Mogi M, Mitani A (2017). Angiopoietin-like protein 2 regulates Porphyromonas gingivalis lipopolysaccharide-induced inflammatory response in human gingival epithelial cells. PLoS One.

[5] Keles Z, Keles G, Avci B, Cetinkaya B, Emingil G (2014). Analysis of YKL-40 acute-phase protein and interleukin-6 levels in periodontal disease. J Periodontol.

[6] Luo W, Wang C, Jin L (2012). Baicalin downregulates Porphyromonas gingivalis lipopolysaccharide-upregulated IL-6 and IL-8 expression in human oral keratinocytes by negative regulation of TLR signaling. PLoS One.

[7] Pahumunto N, Chotjumlong P, Makeudom A, Krisanaprakornkit S, Dahlen G, Teanpaisan R (2017). Pro-inflammatory cytokine responses in human gingival epithelial cells after stimulation with cell wall extract of Aggregatibacter actinomycetemcomitans subtypes. Anaerobe..

[8] Li H, Zhou X, Zhang J (2014). Induction of heme oxygenase-1 attenuates lipopolysaccharide-induced inflammasome activation in human gingival epithelial cells. Int J Mol Med.

[9] Lee H, Lee H, Kim M, Choi Y, Ahn K, Um J, Lee S, Yang W (2017). Angelica dahurica ameliorates the inflammation of gingival tissue via regulation of pro-inflammatory mediators in experimental model for periodontitis. J Ethnopharmacol.

[10] White J (2018). Vitamin D deficiency and the pathogenesis of Crohn's disease. J Steroid Biochem Mol Biol.

[11] Van Belle T, Vanherwegen A, Feyaerts D, De Clercq P, Verstuyf A, Korf H, Gysemans C, Mathieu C (2014). 1,25-Dihydroxyvitamin D3 and its analog TX527 promote a stable regulatory T cell phenotype in T cells from type 1 diabetes patients. PLoS One.

[12] Peppone L, Ling M, Huston A, Reid M, Janelsins M, Puzas J, Kamen C, Del Giglio A, Asare M, Peoples A, Mustian K (2018). The effects of high-dose calcitriol and individualized exercise on bone metabolism in breast cancer survivors on hormonal therapy: a phase II feasibility trial. Support Care Cancer.

[13] Du J, Li R, Yu F, Yang F, Wang J, Chen Q, Wang X, Zhao B, Zhang F (2017). Experimental study on 1,25(OH)2 D3 amelioration of oral lichen planus through regulating NF-κB signaling pathway. Oral Dis.

[14] Du J, Chen Y, Shi Y, Liu T, Cao Y, Tang Y, Ge X, Nie H, Zheng C, Li Y (2015). 1,25-dihydroxyvitamin D protects intestinal epithelial barrier by regulating the myosin light chain kinase signaling pathway. Inflamm Bowel Dis.

[15] De Filippis A, Fiorentino M, Guida L, Annunziata M, Nastri L, Rizzo A (2017). Vitamin D reduces the inflammatory response by Porphyromonas gingivalis infection by modulating human β-defensin-3 in human gingival epithelium and periodontal ligament cells. Int Immunopharmacol.

[16] Garcia M, Hildebolt C, Miley D, Dixon D, Couture R, Spearie C, Langenwalter E, Shannon W, Deych E, Mueller C, Civitelli R (2011). One-year effects of vitamin D and calcium supplementation on chronic periodontitis. J Periodontol.

[17] Stockinger B, Di Meglio P, Gialitakis M, Duarte J (2014). The aryl hydrocarbon receptor: multitasking in the immune system. Annu Rev Immunol.

[18] Ma Y, Wang Q, Yu K, Fan X, Xiao W, Cai Y, Xu P, Yu M, Yang H (2018). 6-Formylindolo(3,2-b)carbazole induced aryl hydrocarbon receptor activation prevents intestinal barrier dysfunction through regulation of claudin-2 expression. Chem Biol Interact.

[19] Engen S, Rørvik G, Schreurs O, Blix I, Schenck K (2017). The oral commensal Streptococcus mitis activates the aryl hydrocarbon receptor in human oral epithelial cells. Int J Oral Sci.

[20] Øvrevik J, Låg M, Lecureur V, Gilot D, Lagadic-Gossmann D, Refsnes M, Schwarze P, Skuland T, Becher R, Holme J (2014). AhR and Arnt differentially regulate NF-κB signaling and chemokine responses in human bronchial epithelial cells. Cell Commun Signal.

[21] Matsunawa M, Akagi D, Uno S, Endo-Umeda K, Yamada S, Ikeda K, Makishima M (2012). Vitamin D receptor activation enhances benzo[a]pyrene metabolism via CYP1A1 expression in macrophages. Drug Metab Dispos.

[22] Li H, Wang Q, Chen X, Ding Y, Li W (2016). Mangiferin inhibits lipopolysaccharide-induced production of interleukin-6 in human oral epithelial cells by suppressing toll-like receptor signaling. Arch Oral Biol.

[23] Deb D, Chen Y, Zhang Z, Zhang Y, Szeto F, Wong K, Kong J, Li Y (2009). 1,25-Dihydroxyvitamin D3 suppresses high glucose-induced angiotensinogen expression in kidney cells by blocking the NF-{kappa}B pathway. Am J Physiol Ren Physiol.

[24] Wang Q, Li H, Xie H, Fu M, Guo B, Ding Y, Li W, Yu H (2013). 25-Hydroxyvitamin D3 attenuates experimental periodontitis through downregulation of TLR4 and JAK1/STAT3 signaling in diabetic mice. J Steroid Biochem Mol Biol.

[25] Jiang Y, Fleet JC (2012). Phorbol esters enhance 1α,25-dihydroxyvitamin D3-regulated 25-hydroxyvitamin D-24-hydroxylase (CYP24A1) gene expression through ERK-mediated phosphorylation of specific protein 3 (Sp3) in Caco-2 cells. Mol Cell Endocrinol.

[26] Pike J, Meyer M (2010). The vitamin D receptor: new paradigms for the regulation of gene expression by 1,25-dihydroxyvitamin D(3). Endocrinol Metab Clin N Am.

[27] Girgis C, Clifton-Bligh R, Mokbel N, Cheng K, Gunton J (2014). Vitamin D signaling regulates proliferation, differentiation, and myotube size in C2C12 skeletal muscle cells. Endocrinology..

[28] Jamali N, Song Y, Sorenson C, Sheibani N (2019). 1,25(OH)2D3 regulates the proangiogenic activity of pericyte through VDR-mediated modulation of VEGF production and signaling of VEGF and PDGF receptors. FASEB Bioadv.

[29] Zhan Y, Samietz S, Holtfreter B, Hannemann A, Meisel P, Nauck M, Völzke H, Wallaschofski H, Dietrich T, Kocher T (2014). Prospective study of serum 25-hydroxy vitamin D and tooth loss. J Dent Res.

[30] Abreu O, Tatakis D, Elias-Boneta A, López Del Valle L, Hernandez R, Pousa M, Palacios C (2016). Low vitamin D status strongly associated with periodontitis in Puerto Rican adults. BMC Oral Health.

[31] Antonoglou G, Suominen A, Knuuttila M, Ylöstalo P, Ojala M, Männistö S, Marniemi J, Lundqvist A, Tervonen T (2015). Associations between serum 25-hydroxyvitamin d and periodontal pocketing and gingival bleeding: results of a study in a non-smoking population in Finland. J Periodontol.

[32] Papathanasiou E, Kantarci A, Konstantinidis A, Gao H, Van Dyke T (2016). SOCS-3 regulates alveolar bone loss in experimental periodontitis. J Dent Res.

[33] Jönsson D, Aggarwal P, Nilsson B, Demmer R (2013). Beneficial effects of hormone replacement therapy on periodontitis are vitamin D associated. J Periodontol.

[34] Kong J, Zhu X, Shi Y, Liu T, Chen Y, Bhan I, Zhao Q, Thadhani R, Li Y (2013). VDR attenuates acute lung injury by blocking Ang-2-Tie-2 pathway and renin-angiotensin system. Mol Endocrinol.

[35] Vogel C, Khan E, Leung P, Gershwin M, Chang W, Wu D, Haarmann-Stemmann T, Hoffmann A, Denison M (2014). Cross-talk between aryl hydrocarbon receptor and the inflammatory response: a role for nuclear factor-κB. J Biol Chem.

[36] Furumatsu K, Nishiumi S, Kawano Y, Ooi M, Yoshie T, Shiomi Y, Kutsumi H, Ashida H, Fujii-Kuriyama Y, Azuma T, Yoshida M (2011). A role of the aryl hydrocarbon receptor in attenuation of colitis. Dig Dis Sci.

[37] Ji T, Xu C, Sun L, Yu M, Peng K, Qiu Y, Xiao W, Yang H (2015). Aryl hydrocarbon receptor activation down-regulates IL-7 and reduces inflammation in a mouse model of DSS-induced colitis. Dig Dis Sci.

[38] Afaq F, Zaid M, Pelle E, Khan N, Syed D, Matsui M, Maes D, Mukhtar H (2009). Aryl hydrocarbon receptor is an ozone sensor in human skin. J Invest Dermatol.

[39] Vogel C, Matsumura F (2009). A new cross-talk between the aryl hydrocarbon receptor and RelB, a member of the NF-kappaB family. Biochem Pharmacol.

[40] Herath T, Darveau R, Seneviratne C, Wang C, Wang Y, Jin L (2013). Tetra-and penta-acylated lipid a structures of Porphyromonas gingivalis LPS differentially activate TLR4-mediated NF-κB signal transduction cascade and immuno-inflammatory response in human gingival fibroblasts. PLoS One.

[41] Elburki M, Rossa CJ, Guimarães-Stabili M, Lee H, Curylofo-Zotti F, Johnson F, Golub L (2017). A chemically modified curcumin (CMC 2.24) inhibits nuclear factor κB activation and inflammatory bone loss in murine models of LPS-induced experimental periodontitis and diabetes-associated natural periodontitis. Inflammation.

[42] Gugliandolo E, Fusco R, D'Amico R, Militi A, Oteri G, Wallace J, Di Paola R, Cuzzocrea S (2018). Anti-inflammatory effect of ATB-352, a H2S -releasing ketoprofen derivative, on lipopolysaccharide-induced periodontitis in rats. Pharmacol Res.

[43] Jensen B, Leeman R, Schlezinger J, Sherr D (2003). Aryl hydrocarbon receptor (AhR) agonists suppress interleukin-6 expression by bone marrow stromal cells: an immunotoxicology study. Environ Health.

[44] Tian Y, Ke S, Denison M, Rabson A, Gallo M (1999). Ah receptor and NF-kappaB interactions, a potential mechanism for dioxin toxicity. J Biol Chem.

[45] Verma R, Jung J, Kim J (2014). 1,25-Dihydroxyvitamin D3 up-regulates TLR10 while down-regulating TLR2, 4, and 5 in human monocyte THP-1. J Steroid Biochem Mol Biol.

[46] Jiang J, Shi D, Zhou X, Yin L, Feng L, Jiang W, Liu Y, Tang L, Wu P, Zhao Y (2015). Vitamin D inhibits lipopolysaccharide-induced inflammatory response potentially through the toll-like receptor 4 signalling pathway in the intestine and enterocytes of juvenile Jian carp (Cyprinus carpio var. Jian). Br J Nutr.

[47] Chen W, Yan Q, Yang H, Zhou X, Tan Z (2019). Effects of restrictions on maternal feed intake on the immune indexes of umbilical cord blood and liver toll-like receptor signaling pathways in fetal goats during pregnancy. J Anim Sci Biotechnol.

[48] Salazar F, Hall L, Negm O, Awuah D, Tighe P, Shakib F, Ghaemmaghami A (2016). The mannose receptor negatively modulates the toll-like receptor 4-aryl hydrocarbon receptor-indoleamine 2,3-dioxygenase axis in dendritic cells affecting T helper cell polarization. J Allergy Clin Immunol.

